# What I Learned from Analyzing Accurate Mass Data of 3000 Supporting Information Files

**DOI:** 10.1021/acs.orglett.4c03458

**Published:** 2024-12-19

**Authors:** Mathias Christmann

**Affiliations:** Institute of Chemistry and Biochemistry, Freie Universität Berlin, Takustrasse 3, 14195 Berlin, Germany

## Abstract

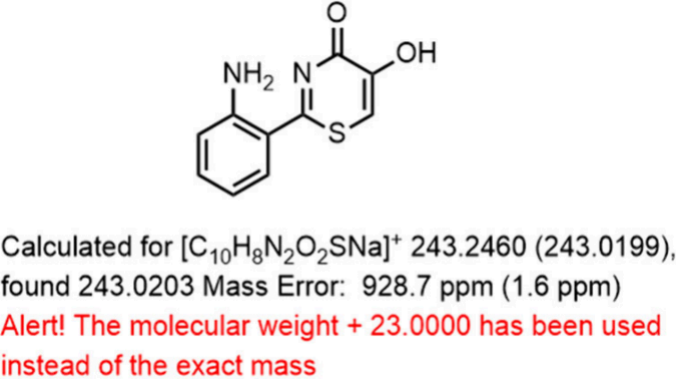

A Python script for the systematic, high-throughput analysis of accurate mass data was developed and tested on more than 3000 Supporting Information (SI) PDFs from *Organic Letters*. For each SI file, quadruplets of molecular formula, measured ion, e.g., [M + Na]^+^, and reported calculated and found masses were extracted and analyzed. Interestingly, only 40% of the files containing readable accurate mass data were both internally consistent and in compliance with *The ACS Guide to Scholarly Communication*. The analysis revealed unexpected errors and provided actionable advice on how to improve data quality.

The rapid growth of the scientific literature is driving the need for automated tools to efficiently extract, process, and analyze critical data. In chemistry, data sets documenting the synthesis of new chemical compounds typically consist of detailed preparation procedures, accompanied by characterization data to confirm the purity and structural integrity. Experimental sections have traditionally been written by humans for humans to facilitate replication and validation, as well as to allow verification of the work through visual inspection. In the age of digitization and automation, ongoing efforts aim to make natural language synthesis instructions machine-readable and -actionable,^1^ leveraging robotic technologies^2,3^ and enabling self-optimization.^4^ In today’s data-driven chemistry landscape, innovations that generate or curate high-quality, structured data sets are as essential as traditional experimental advancements.^5^ Evaluating experimental data from research articles and Supporting Information has become an increasingly time-consuming task for authors, reviewers, and editors alike. In 2004, Goodman et al. developed an applet to semiautomatically check various characterization data copied and pasted from manuscripts.^6^ Detailed tests were conducted on 10 papers, and a further survey was conducted on 100 randomly selected data paragraphs of 50 papers. The conclusion was that “preliminary tests with this program demonstrate that refereed and published experimental data are highly accurate, but errors are still occasionally perpetuated”. While tools such as the experimental data checker can help improve data quality, their focus on individual errors limits the ability to gain broader insights into error patterns. The following research takes a closer look at the nature of errors in experimental chemistry papers using a single metric: accurate mass measurements using high-resolution mass spectrometry (HRMS). Accurate mass measurements (AMMs) confirm the proposed molecular formula and can be used to distinguish elemental compositions with similar nominal masses. The recorded data enables automatic verification of internal consistency. It was anticipated that a high-throughput review of the accurate mass measurement data of more than 3000 SI files might reveal patterns not visible through random sampling. To achieve this goal, a Python script was developed to perform a large-scale analysis of the data by systematically addressing the following tasks:

1. locate all PDFs within a given folder
2. locate and extract all accurate mass data from each PDF
3. for each measurement, recalculate the accurate mass of the measured ion
4. calculate deviations of measured, calculated, and recalculated masses (in parts per million)
5. print a one-line analysis of each measurement highlighting unusual deviations
6. in cases of internal inconsistencies, provide a plausible explanation or, if possible, a solution to the problem
7. create a summary for all files investigated

Before the results of the automated screening are discussed, it is important to note that the Python script can check for only internal consistency; i.e., it is beyond the scope of this analysis to verify whether a molecular formula corresponds to the chemical structure. In addition, the conventions for reporting HRMS measurements in experimental sections need to be addressed. These vary from journal to journal in terms of acceptable deviations and the presentation of results. *The Journal of Organic Chemistry*’s author guidelines state that for HRMS measurements, “The reported molecular formulas and Calcd values should include any added atoms (usually H or Na). The ionization method and mass analyzer type (e.g., Q-TOF, magnetic sector, or ion trap) should be reported. *The ACS Guide to Scholarly Communication* format for reporting accurate mass data is HRMS (ESI/Q-TOF) m/z: [M + Na]^+^ Calcd for C_13_H_17_NO_3_Na 258.1101; Found 258.1074.”

To demonstrate how the Python script works, a synthetic SI PDF was generated and placed in the Downloads folder. Including the paragraph quoted above within that file results in the output shown in Figure 1 (section a). The exact mass is followed by the recalculated value shown in parentheses, while the mass errors refer to the reported calculated and recalculated masses (in parentheses) in relation to the found mass. In the example presented above, the respective mass errors colored red exceed an allowed arbitrary threshold of 10 ppm. Tolerated deviations can vary between the journals.

**Figure 1:**
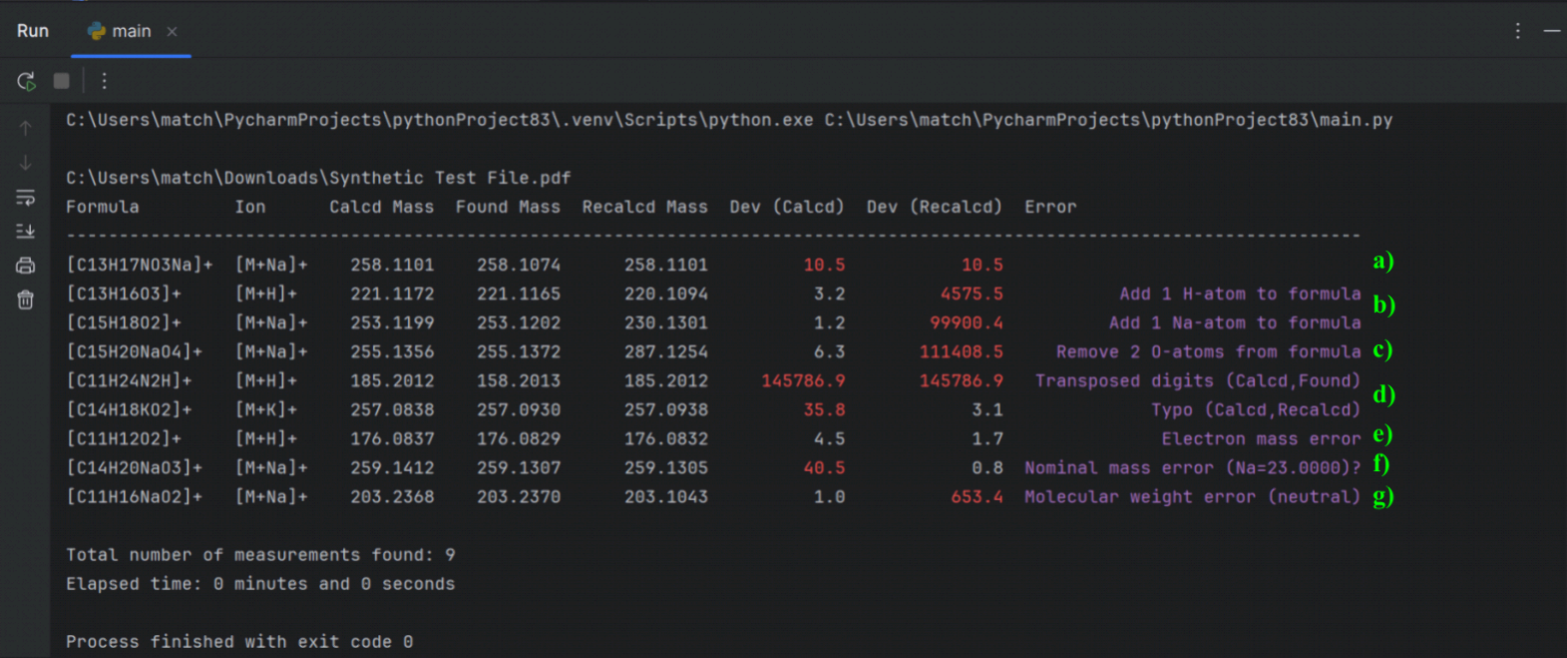
Automatic evaluation of a synthetic test PDF using the Python script in the PyCharm IDE. (a) The parts per million deviation is above the threshold. (b) The added atoms ([M + H] and [M + Na]) are not included in the formula. (c) Incorrect formula. (d) Typographical errors. (e) Mass calculated for the neutral molecule. (f) Nominal mass added. (g) Molecular weight used instead of exact mass.

A second type of internal inconsistency occurs when the formula of the measured ion does not match the calculated mass. Discrepancies often arise when added atoms, such as [M + H] or [M + Na], have not been included in the formula. While the root of this error is primarily a matter of convention (see author guidelines above), there are strong arguments for stating the formula of the actual ion being measured in HRMS. Reporting only the molecular formula in cases of [M + H] or [M + Na] measurements complicates verification and invites errors, as it necessitates additional steps such as adding atoms (or, worse, adding the mass of atoms) to obtain the displayed calculated mass. The Python script identifies missing atoms and suggests a formula that fits the calculated mass (Figure 1, section b).

Additionally, errors in the molecular formula may arise from workflows involving redundant human interventions. The accurate mass measurement itself includes an internal control mechanism. An incorrect molecular formula will not lead to a matching mass measurement. Errors occur if an incorrect formula is paired with the calculated and measured masses after the measurement. This can happen due to incorrect formula transfer (e.g., mistyping) or by pairing the numeric data with a newly generated formula. The script catches these mistakes and suggests a formula that does fit the calculated and measured masses by modulating the atomic composition of the given incorrect molecular formula (Figure 1, section c).^7^

Similar human-in-the-loop errors can occur when manually retyping numerical values from a printed report instead of directly transferring them into the SI. Common mistakes include transposition errors, where two adjacent digits are swapped, and substitution errors, such as typing “8” instead of “9” due to the proximity of keys on the keyboard (see Figure 1, section d).

After addressing formula errors and typos, we now turn to inconsistencies resulting from miscalculations. A common, albeit subtle, numerical discrepancy (in the low parts per million range) is observed when the exact mass is calculated for a neutral molecule, while the measured mass corresponds to a charged species, typically a cation (Figure 1, section e). Significantly larger mass errors in adducts ([M + H] and [M + Na]) arise if the nominal mass^8^ of the added atom (1.0000 for H or 23.0000 for Na) is used instead of its precise isotopic mass (1.0078 for H or 22.9898 for Na) (Figure 1, section f). Unlike elements such as sodium (Na) and fluorine (F), which are monoisotopic, most elements, such as carbon (C) and hydrogen (H), are polyisotopic. In compounds containing these polyisotopic elements, confusion between the molecular weight (MW) and monoisotopic mass usually results in significant errors (Figure 1, section g). This example also illustrates a situation in which an apparent miscalculation is misleadingly validated by matching measurements. In rare instances, certain isotopic compositions result in the molecular weight and exact mass being very similar or identical. For example, the exact mass of C_1__1_H_2__2_BN_2_^+^ is 265.1871, while the molecular weight of C_1__1_H_2__2_BN_2_ is 265.1870.

Efficient extraction and processing of large data sets can enable meta-analyses that reveal hidden patterns and trends. Recognizing that *Organic Letters* is committed to delivering high-quality Supporting Information,^9^ we initiated an analysis of more than 3000 SI PDFs from the journal. To foster a discussion on how to further improve data quality, Table 1 summarizes a screening of all SI PDFs from 2023 and 2024 (issues 1–36), comprising 3028 files and totaling 26.3 GB of data.

**Table 1:** Screening of the Supporting Information of *Organic Letters* (2023 to the present) for Accurate Mass Measurements (AMMs)

	*Organic Letters* 2023, issues 1–51	*Organic Letters* 2024, issues 1–36
SI PDF files	1677 (14.5 GB)	1351 (11.8 GB)
files with AMM data	1618 (96%)	1294 (96%)
files without AMM errors^a^	662 (41%)	519 (40%)
AMMs	56 134	45 749
AMM errors	16 955 (30%)	12 694 (28%)
AMM errors (without e^–^)	4773 (9%)	4622 (10%)
molecular weight errors	17	25
nominal mass errors	91	147
electron mass errors (e^–^)	12 182	8074
transposed digits	9	6
typographical errors	53	47
one H atom added	1617	1362
one Na atom added	679	393
one O atom added	21	23
one C atom added	10	8
two H atoms added	7	8
two O atoms added	7	6
one H atom removed	154	241
one Na atom removed	9	8
one CH_2_ group removed	3	4

a Excluding electron mass errors.

All calculations were performed on a personal computer with no need for any data to leave the device. On a laptop computer (EliteBook 840 G8, Intel i5 @ 2.4 GHz), scanning a single SI PDF takes <1 s and checking a whole volume of *Organic Letters* SI PDFs takes ∼15 min. The script demonstrated a high accuracy rate (>99%), successfully identifying AMM data in >95% of the analyzed files. The remaining <5% largely comprised files that lacked AMM data altogether (e.g., those related to computational studies).

Among the files with AMM data, only ∼40% fully adhered to the journal guidelines. The predominant minor deviation observed was the calculation of the exact mass for the neutral molecule rather than for the charged species. The second most frequent error involved the omission of added atoms (e.g., [M + H] and [M + Na]) in the molecular formula. Cases in which formulas included incorrect or missing atoms (such as O, F, and Cl) or groups (e.g., CH_2_) were swiftly detected and corrected. Similarly, simple typographical errors were easily identified and addressed.

In addition to these minor oversights, the script uncovered 280 significant errors (molecular weight errors and nominal weight errors), which were often rooted in fundamental misunderstandings of how to correctly calculate exact masses. In some cases, unusual discrepancies between measured and miscalculated masses were simply overlooked, while in others, the measurements appeared to validate the incorrect calculations. It is hoped that this script will save authors and reviewers considerable time and effort in identifying and correcting such errors before publication.

What can we learn from this? To enhance data quality, it is essential to implement automated protocols that take the human out of the loop in data handling postmeasurement, thus reducing the risk of manual errors. Additionally, adhering to a journal’s conventions for presenting data is essential to reduce ambiguity and facilitate verification. The problem can be approached from both ends by both optimizing for machine readability^10^ and by devising tools^11^ that can help to translate chemical language and representations such as chemical drawings.

In the realm of chemical education, it is important to emphasize the distinctions among the nominal mass, exact mass, molecular weight, and mass differences between charged and uncharged species ([M] vs [M]^+^). Figure 2 shows the magnitude of the error relative to the mass of the [M + Na]^+^ cation depending on how the exact mass was miscalculated.

**Figure 2:**
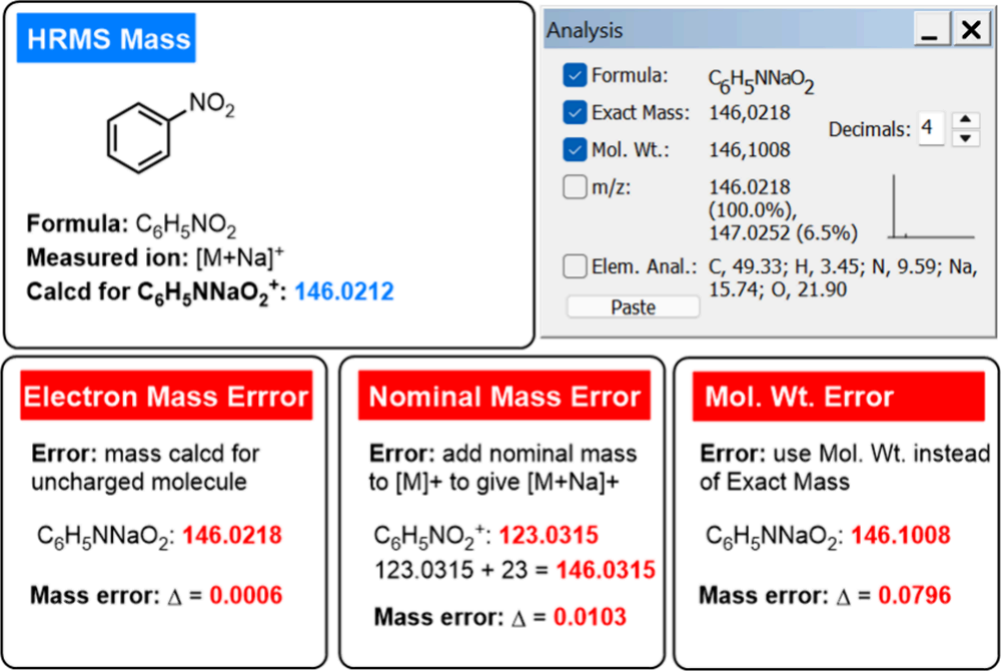
Examples of calculating and miscalculating the exact mass using ChemDraw.

Although this author had no prior coding experience with Python, this script was developed in a relatively short time frame by following a 4-hour Python tutorial, leveraging large language models (LLMs) such as ChatGPT, Gemini, and Claude for code generation and utilizing existing Python libraries like Molmass.^12^ By releasing this script as open-source software, I hope to contribute to improving the quality and reliability of scientific data and inspire other data-driven approaches.

## Data Availability

The Python script and the test file can be accessed at https://github.com/match22lab/HRMS-Checker-2.0.
